# Acute mucocutaneous syndrome and hemophagocytic lymphohistiocytosis following COVID-19 with prominent emperipolesis

**DOI:** 10.1016/j.jdcr.2026.05.034

**Published:** 2026-05-29

**Authors:** Kylie S. Andonian, John Strasswimmer

**Affiliations:** a Charles E. Schmidt College of Medicine, Florida Atlantic University, Boca Raton, Florida; b Dermatology Associates of the Palm Beaches, Delray Beach, Florida

**Keywords:** COVID-19, cutaneous manifestations, emperipolesis, hemophagocytic lymphohistiocytosis (HLH), hemophagocytosis

## Background

Although initially characterized as a primary respiratory illness, COVID-19 is now well recognized as a multisystem inflammatory disorder with neurologic, vascular, immunologic, and dermatologic involvement.^1^ Acute COVID-related cutaneous syndromes have been identified in patients of all ages and encompass a range of clinical morphologies, including vesicular, urticarial, morbilliform, petechial, livedo reticularis, chilblain-like, and vasculitic patterns.^2^ In some cases, cutaneous presentations may precede or accompany severe systemic inflammation.^1^

Emperipolesis describes the presence of an intact cell within the cytoplasm of another cell, in contrast to hemophagocytosis, in which engulfed hematopoietic cells are phagocytosed and destroyed by activated macrophages.^3^ Although hemophagocytosis is a diagnostic feature of HLH, emperipolesis is not a typical reported marrow finding.

The purpose of this case report is to identify a rare, life-threatening case of acute mucocutaneous syndrome with secondary HLH following COVID-19, in which prominent emperipolesis was identified on bone marrow examination.

## Case presentation

**Fig 1:**
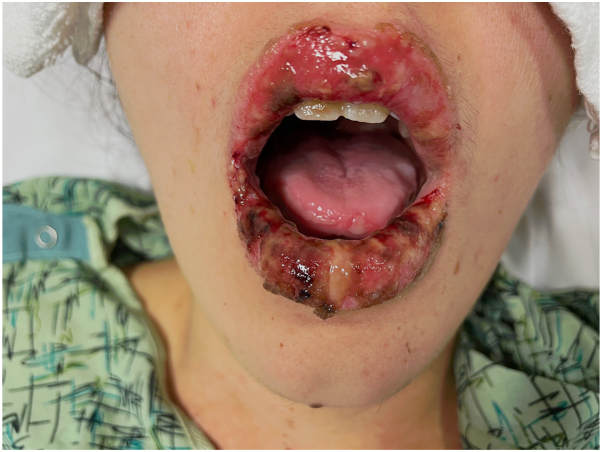
Confluent hemorrhagic mucosal erosions of the lips.

A 23-year-old previously healthy female presented to the emergency department with a 1-week history of progressively worsening fever, rash, and orofacial swelling, requiring intubation. After she mentioned feeling ill 2 weeks prior, a positive Immunoglobulin M antibody indicated acute COVID-19 infection. Physical examination was notable for head and neck lymphadenopathy and periorificial edema. Cutaneous examination revealed morbilliform eruption of the trunk, hemorrhagic oral erosions, mild edema and tenderness of the hand joints, pseudo-chilblains with livedo racemosa of the palmar and plantar skin, and bilateral conjunctivitis (Fig 1).

Initial laboratory evaluation demonstrated pancytopenia and elevated hepatic transaminases. On hospital day 2, the patient developed acute encephalopathy requiring intubation. Given concern for infectious meningoencephalitis, broad-spectrum antimicrobial therapy was initiated, including doxycycline, ceftriaxone, ampicillin, and acyclovir. Computed tomography of the chest and magnetic resonance imaging of the brain demonstrated bilateral mastoiditis and chronic sinusitis.

An extensive infectious workup was negative, including testing for HSV, HIV, CMV, TB, EBV, syphilis, Bartonella, measles, mumps, Lyme disease, toxoplasmosis, Cryptococcus, Mycoplasma, West Nile virus, dengue, and viral hepatitis. Bloodwork was notable for elevated ferritin.

**Fig 2:**
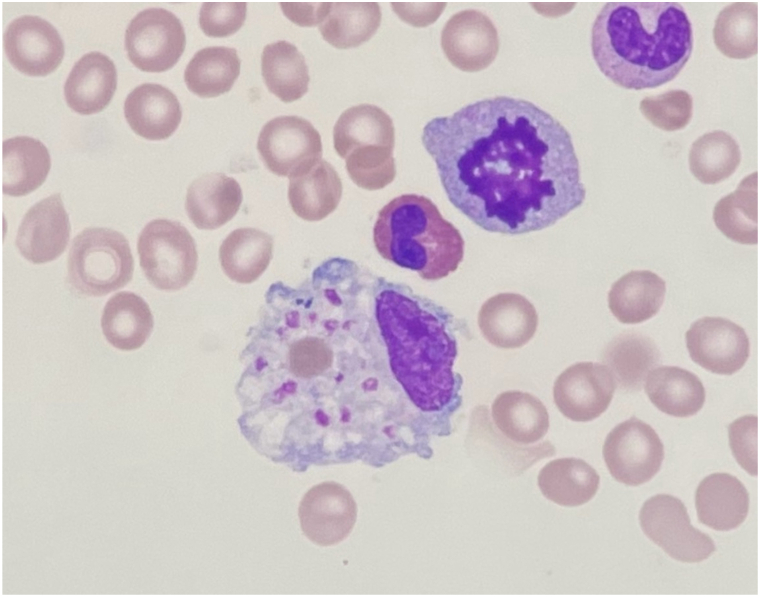
High-power bone marrow aspirate smear demonstrating prominent emperipolesis.

Bone marrow biopsy revealed both hemophagocytosis and emperipolesis, the latter of which describes intact cells such as erythrocytes, lymphoid cells, myeloid cells, neutrophils, or mature red blood cells within the cytoplasm rather than phagocytosed cellular debris (Fig 2). According to the 2004 HLH diagnostic guidelines, the patient met 7 of 8 criteria for HLH, including fever (38.4 °C), cytopenias, hypertriglyceridemia (526 mg/dL), hemophagocytosis in bone marrow aspirate smear, decreased natural killer cell activity, elevated ferritin (2329-5314 ng/mL), and elevated soluble interleukin-2-receptor levels.^4^ The H-Score was calculated at 191, corresponding to a high probability of HLH.^4^

Treatment with etoposide was initiated following oncology consultation, resulting in gradual improvement over 6 months.

## Conclusion

We report a previously healthy, young female who developed acute mucocutaneous syndrome and HLH secondary to COVID-19, with bone marrow biopsy revealing hemophagocytosis and prominent emperipolesis. This case is unique in that it documents the rare association between emperipolesis and severe hyperinflammatory disease following a COVID-19 infection. This occurred in January of 2021, which was the peak of the COVID-19 pandemic, although prior to the availability of vaccinations.

The wide range of cutaneous manifestations of COVID-19 each demonstrate distinct immunopathologic mechanisms.^1^ In this patient, the combination of morbilliform eruption, hemorrhagic mucosal erosions, conjunctivitis, acral livedoid changes, and inflammatory joint involvement revealed an acute systemic inflammatory response. The prominent lip mucositis and conjunctivitis raise consideration of reactive infectious mucocutaneous eruption in the differential diagnosis. However, the patient’s broader multisystem hyperinflammatory presentation, including pancytopenia, hyperferritinemia, neurologic deterioration, and bone marrow hemophagocytosis, favored COVID-19-associated secondary HLH as the unifying diagnosis.^5^ Since the dermatologic findings preceded the neurologic deterioration and diagnosis of HLH, this reinforces the idea that acute mucocutaneous syndromes in COVID-19 may serve as an early indication of systemic immune collapse.

In patients with recent or active COVID-19 infection presenting with severe mucocutaneous disease, cytopenias, elevated ferritin levels, and neurologic decline, clinicians should maintain a high index of suspicion for secondary HLH.^4^ In reporting this case, recognition of emperipolesis may serve as an additional pathologic phenomenon supporting a diagnosis of severe immune dysregulation in COVID-19. The patient’s response to etoposide further supports the immune-mediated dysregulation of this disease process and underscores the significance of bone marrow biopsy and early aggressive immunosuppression in preventing irreversible organ damage and mortality.

## Conflicts of interest

None disclosed.
